# Supplementary material for: Differences in Soil Fungal Communities between Forested Reclamation and Forestry Sites in the Alberta Oil Sands Region
Source: J Fungi (Basel). 2023 Nov 16;9(11):1110. doi: 10.3390/jof9111110 (PMC10672713; doi:10.3390/jof9111110)
Supplement: Supplementary file 1 [file jof-09-01110-s001.zip › jof-2660199-supplementary.docx]

**Supplementary Material**

*Text S1, S2, S3; Tables S1 to S10; Figures S1 to S5.*

**Journal of Fungi**

Trofymow, Shay, Tomm, Bérubé, Ramsfield

Differences in soil fungal communities between forested reclamation and forestry sites

in the Alberta oil sands region.

**S1)** *Disturbance effects on mycorrhizal and macro- and micro-saprotrophic fungal functional groups*

Effects of disturbance vary with fungal functional group. The functional groups most sensitive to disturbance are the mycorrhizal fungi (for example, ectomycorrhizae [EM], arbuscular mycorrhizae and ericoid mycorrhizae; Zak, 1992). In exchange for photosynthate from their host plant, they increase nutrient uptake to their host plant through their microscopic hyphal network and, in some cases, increase mineralization of nitrogen, phosphorus and some other nutrients from inorganic and organic forms. Published lists of EM fungi and associated tree species reveal that about 225 EM fungal species are associated with 12 principal boreal tree species found in North America (see below for supplementary list of species), with 89 EM species associated with only one tree species, 25 with two tree species, 14 with three tree species, seven with four or five tree species, and only one truly cosmopolitan EM fungal species, *Cenococcum graniforme* (Sowerby) Ferd. & Winge, associated with 11 tree species.

By contrast, macro- and microsaprotrophic fungi, also referred to as saprobes, use extracellular enzymes to digest and decay dead organic material such as wood (so-called lignicolous species) and plant and animal litter (Carroll and Wicklow, 1992) and grow and disperse as long as a sufficient substrate is present. Saprobic macrofungi common to boreal forests include species of Mycena, Marasmius, Galerina, Trametes, Aleuria, and Xylaria genera. Saprobic macrofungi are not as numerous as microfungi and tend to be specific to certain substrates (Lumley et al., 2001).

Pathogenic macrofungi are natural components of forest ecosystems; most of what is known about these fungi in forests is limited to pathogens affecting trees, usually the dominant form of vegetation (Durall et al., 2005). Pathogenic fungi have several roles in plant communities (Winder and Shamoun, 2006), serving as essential drivers of succession when they kill trees and induce gap formation but becoming pests when they interfere with forest management objectives (Castello et al., 1995; Durall et al., 2005). Fungal pathogens affecting boreal tree species are found primarily in the Basidiomycota and Ascomycota divisions. In Canada, such pathogens include native fungal species causing root and stem decay (root disease; e.g., *Armillaria ostoyae* (Romagn.) Herink, *Onnia tomentosa* (Fr.) P. Karst., *Heterobasidion annosum sensu lato*, *Fomitopsis pinicola* (Sw.) P. Karst., and several Phellinus spp.) (Laflamme, 2010; Morrison et al., 1992), stem rusts (e.g., *Chrysomyxa spp.,* *Cronartium spp.* and *Endocronartium harknessii* (J.P. Moore) Y. Hirats.), and needle/foliar diseases (e.g., *Dothistroma septosporum* (Dorogin) M. Morelet).

Brandt, J.P., 2009. The extent of the North American boreal zone. Environ. Rev. 17: 101-161.

Bossenmaier, E.F., 1997. Mushrooms of the Boreal Forest. University Extension Press, University of Saskatchewan, Saskatoon, Saskatchewan.

Chen, M.M., 2003. Fungi of the Alaskan Inland Ecosystem, in: Chen, M.M., Forest Fungi Phytogeography: Forest Fungi Phytogeography of China, North America, and Siberia and International Quarantine of Tree Pathogens, Pacific Mushroom Research and Education Centre, Sacramento, California, pp. 174-227.

Farrar, J.L., 1995. Trees in Canada. Natural Resources Canada, Canadian Forest Service, Ottawa, Ontario, co-published by Fitzhenry and Whiteside Limited, Markham, Ontario.

Lumley, T.C., Gignac, L.D., Currah, R.S., 2001. Microfungus communities of white spruce and trembling aspen logs at different stages of decay in disturbed and undisturbed sites in the boreal mixedwood region of Alberta. Can. J. Bot. 79, 76-92.

Trappe, J.M., 1962. Fungus associates of ectotrophic mycorrhizae. Bot. Rev. 28, 538-606.

Winder, R.S., Shamoun, S.F., 2006. Forest pathogens: friend or foe to biodiversity? Can. J. Plant Pathol. 28, S221-S227.

**S2)** *List of fungal species in boreal forests*

Ectomycorrhizal fungal species associated with principal boreal forest tree species (after Brandt, 2009 and Farrar, 1995) in North America. Numbers following each EM fungal species refers to source reference in which it was included: 1 – Trappe (1962), 2 – Chen (2003), and 3 – Bossenmaier (1997).

*Picea glauca* – white spruce

*Amanita ceciliae 2, Amanita muscaria 3, Boletus edulis 3, Cenococcum graniforme 1, Cortinarius alboviolaceous 3, Cortinarius brunneus 2, Cortinarius semisanguineus 3, Cortinarius violaceous 3, Gomphus clavatus 3, Hebeloma sinapizans 3, Hydnellum caeruleum 3, Hydnum fuscoindicum 3, Hydnum imbricatum 3, Hydnum scabrosum 3, Hygrophorus chrysodon 3, Hygrophorus discoideus 3, Hygrophorus erubescens 3, Hygrophorus olivaceoalbus 3, Hygrophorus piceae 3, Inocybe sororia (mixed forest) 3, Laccaria laccata 23, Lactarius deliciosus var. deliciosus 23, Lactarius resimus 3, Lactarius rufus 3, Lactarius scrobiculatus 3, Leccinum aurantiacum 2, Russula emetica 2, Russula nigricans 2, Russula rosacea 2, Sarcodon imbricatus 2, Suillus cavipes 3, Suillus grevillei 3, Suillus luteus, 2, Thelephora terrestris (several habitats) 3, Tricholoma inamoenum 3, Tricholoma saponaceum 3, Tricholoma vaccinum 23, Tricholoma virgatum 3, Tricholoma zelleri 3,* Genera *2: Clitocybe, Gomphidius, Suillus, Amanita, Agaricus, Lactarius*

*Picea mariana* – black spruce

*Cenococcum graniforme 1, Chantherellus (Craterellus) tubaeformis 3, Gomphidius septentrionalis 1, Leccinum alaska 2, Polyporus circinatus 1, Rozites caperata 3, Russula emetica 2, Suillus grevillei 2, Suillus luteus 2,* Genera *2: Clitocybe*

*Populus tremuloides* – trembling aspen

*Amanita fulva 3, Amanita muscaria 3, Amanita vaginata 3, Boletus edulis 3, Boletus piperatus 3, Cenococcum graniforme 1, Cortinarius alboviolaceous 3, Cortinarius trivialis 3, Gomphus clavatus 23, Hebeloma sinapizans 3, Hygrophorus eburneus (mixed forest) 3, Inocybe sororia (mixed forest) 3, Lactarius controversus 3, Lactarius torminosus 3, Leccinum aurantiacum 12, Leccinum insigne 23, Leccinum scabrum 1, Russula chamaeleontina 3, Russula emetica 2, Russula xerampelina 2, Tricholoma saponaceum 3,* Genera *2: Hydnellum, Amanita, Agaricus, Russula, Leccinum, Clitocybe, Gomphus, Clavariadelphus, Sarcodon, Laccaria*

*Larix laricina* - tamarack

*Cenococcum graniforme 1, Chantherellus (Craterellus) tubaeformis 3, Gomphidius maculatus 1, Hygrophorus pseudolucorum 1, Hygrophorus speciosus 3, Inocybe sororia (mixed forest) 3, Suillus cavipes 3, Suillus grevillei 23*

*Abies balsamea* – balsam fir

*Cenococcum graniforme 1, Cortinarius brunneus 2, Suilus granulatus 1*, Genera 2: *Lactarius, Suillus, Hygrophorus, Clitocybe, Tricholoma, Russula, Agaricus*

*Abies lasiocarpa –* subalpine fir

*Cenococcum graniforme 1, Russula delica 1*

*Pinus contorta var. latifolia –* lodgepole pine

*Amanita muscaria 1, Amanita pantherina 1, Amanita vaginata 1, Brauniellula leucosarx 1, Brauniellula nancyae 1, Cenococcum graniforme 1, Cortinarius croceofolius 1, Gomphidius rutilus 1, Gomphidius smithii 1, Gomphidius vinicolor 1, Hygrophorus gliocyclus 1, Laccaria laccata 1, Lactarius deliciosus 1, Leccinum aurantiacum 12, Rhizopogon luteolus 1, Russula delica 1, Suillus granulatus 1, Suillus piperatus (=Boletus piperatus) 1, Suillus ruber (=Boletus ruber) 1, Suillus subaureus 1, Suillus subluteus 1, Suillus tomentosus 1, Suilus bovinus 1, Tricholoma flavovirens 1, Tricholoma ponderosum (= Tricholoma magnivelare) 1, Tricholoma vaccinum 2*

*Pinus banksiana* – jack pine

*Amanita pantherina 1, Amanita rubescens 1, Boletus edulis 3, Boletus piperatus 3, Cenococcum graniforme 1, Chantharellus cibarius 3, Cortinarius cinnamomeus 3, Cortinarius semisanguineus 3, Gomphidius vinicolor 1, Hebeloma crustuliniforme 3, Hydellum pineticola 3, Hydnum imbricatum 3, Hydnum scabrosum 3, Hygrophorus chrysodon 3, Hygrophorus eburneus (mixed forest) 3, Hygrophorus purpurascens 3, Inocybe sororia (mixed forest) 3, Laccaria laccata 3, Lactarius deliciosus (varied habitats) 3, Lactarius indigo 3, Lactarius quietus 1, Lactarius rufus 3, Lactarius scrobiculatus 3, Lactarius uvidus 3, Leccinum insigne 3, Phellodon tomentosus 3, Russula brevipes 3, Russula densifolia 3, Russula nigricans 3, Russula xerampelina 3, Suillus brevipes 3, Suillus cavipes 3, Suillus cothurnatus 1, Suillus granulatus 3, Suillus grevillei 3, Suillus luteus 1, Suillus tomentosus 13, Suillus umbonatus 3, Thelephora terrestris (several habitats) 3, Tricholoma caligatum 3, Tricholoma flavovirens 3, Tricholoma imbricatum 3, Tricholoma magnivelare 3, Tricholoma portentosum 3, Tricholoma saponaceum 3, Tricholoma vaccinum 3, Tricholoma virgatum 3, Tricholoma zelleri 3,* Genera 3: *Chroogomphus, Rhizopogon*

*Pinus strobus –* eastern white pine

*Amanita caesaria 1, Amanita citrina 1, Amanita flavorubescens 1, Amanita muscaria 1, Amanita rubescens 1, Amanita verna 1, Boletinus pictus 1, Boletus rubellus 1, Calvatia saccata 1, Cantharellus cibarius 1, Cenococcum graniforme 1, Clitocybe diatreta 1, Clitocybe odora 1, Collybia butyracea 1, Gomphidius nigricans 1, Gomphidius septentrionalis 1, Gyrodon merulioides 1, Gyroporus castaneus 1, Inocybe eutheles 1, Lactarius chrysorrheus 1, Lactarius deliciosus 1, Lactarius rufus 1, Lepiota rhacodes 1, Lepista nuda (=Lepista personata) 1, Lycoperdon gemmatum 1, Ramaria ochraceovirens 1, Russula lepida 1, Scleroderma aurantium 1, Suillus americanus 1, Suillus bovinus 1, Suillus brevipes 1, Suillus granulatus 1, Suillus luteus 1, Suillus placidus 1, Suillus punctipes 1, Suillus subluteus 1, Tylopulus felleus 1, Xerocomus subtomentosus 1*

*Betula papyrifera –* white birch

*Amanita fulva 3, Amanita muscaria 2, Boletus edulis 2, Cenococcum graniforme 1, Cortinarius brunneus 2, Gomphus clavatus 3, Hygrophorus eburneus (mixed forest) 3, Inocybe sororia (mixed forest) 3, Lactarius torminosus 3, Leccinum aurantiacum 2, Leccinum insigne 2, Leccinum scabrum 13, Russula emetica 2, Russula rosacea 2, Suillus luteus 2,* Genera 2: *Lactarius, Hebeloma, Russula, Agaricus, Clitocybe, Fuscoboletinus*

*Picea engelmannii –* Engleman spruce

*Cenococcum graniforme 1, Amanita pantherina 1, Lactarius deliciosus 1, Russula delica 1, Russula emetica 1, Suillus ruber 1*

*Salix sp. –* willows

*Boletus edulis 3, Boletus piperatus 3, Lactarius aspideoides 3, Tricholoma cingulatum 3*

**S3)** *Details of 454 pyrosequencing methods*

For 454 pyrosequencing, each reaction mixture contained 25 µg of bovine serum albumin, 2.5 µL of polymerase chain reaction (PCR) buffer 10× concentrate (Invitrogen, Carlsbad, CA, USA), 75 mmol/L magnesium chloride, 200 µmol/L of each dNTP, 12.5 µmol/L of each of the forward and reverse primers, 0.2 µL of Platinum® Taq DNA polymerase (Invitrogen), and 5 µL of genomic DNA. The final volume was adjusted to 25 µL using 11.8 µL of UltraPure™ water (Invitrogen). The PCR cycle parameters consisted of initial denaturation at 95 °C for 2 min, followed by 30 cycles of denaturation at 94 °C for 45 s each, annealing at 50 °C for 45 s, extension at 72 °C for 45 s, and a final extension step at 72 °C for 10 min.

**Table S1:** Forest floor depth and nutrient concentrations in forest floors and mineral soils along transects in jack pine stands at the Gateway Hill and Fort Chipewyan road areas.

|  |  |  |  | **Forest floor** | | | | |  | **Mineral soils** | | | |
| --- | --- | --- | --- | --- | --- | --- | --- | --- | --- | --- | --- | --- | --- |
| **Area** | **Site** | **Tree species** | **Replicate** | **Depth (cm)** | **NO_3_^-^**  **(μg g^-1^)** | **NH_4_^+^**  **(μg g^-1^)** | **Total N (%)** | **P**  **(μg g^-1^)** |  | **NO_3_^-^**  **(μg g^-1^)** | **NH_4_^+^**  **(μg g^-1^)** | **Total N (%)** | **P**  **(μg g^-1^)** |
| Gateway | M2 | Jack pine | 1 | 2.0 | 2.81 | 41.5 | 0.771 | 0.54 |  | 2.11 | 16.0 | 0.092 | 0 |
| Hill |  |  | 2 ^†^ | 1.5 | 2.37 | 50.7 | 0.114 | 19.00 |  | 3.89 | 16.9 | 0.971 | 0.30 |
|  |  |  | 3 | 2.0 | 13.90 | 132.2 | 1.110 | 12.50 |  | 2.81 | 15.1 | 0.144 | 0 |
|  | M7 | Jack pine | 1 | 5.0 | 50.90 | 46.9 | 0.922 | 17.90 |  | 5.29 | 15.3 | 0.182 | 0.57 |
|  |  |  | 2 ^†^ | 2.7 | 97.80 | 79.6 | 0.841 | 47.00 |  | 6.89 | 11.7 | 1.150 | 2.16 |
|  |  |  | 3 | 2.5 | 144.50 | 102.3 | 0.172 | 49.10 |  | 9.23 | 12.7 | 0.187 | 0.25 |
| Fort | TR1B2 | Jack pine | 1 ^†^ | 0 | NA | NA | 0.170 | NA |  | 2.28 | 16.1 | 0.057 | 35.30 |
| Chipewyan |  |  | 2 | 0.1 | NA | NA | 0.181 | NA |  | 3.34 | 14.4 | 0.065 | 43.00 |
|  |  |  | 3 | 0 | NA | NA | 0.353 | NA |  | 4.05 | 13.1 | 0.057 | 37.70 |
|  | TR1P | Jack pine | 1 | 0.7 | 2.83 | 20.6 | 0.400 | 102.00 |  | 2.39 | 14.1 | 0.063 | 33.50 |
|  |  |  | 2 ^†^ | 1.5 | 3.86 | 22.3 | 0.837 | 63.90 |  | 3.63 | 14.6 | 0.054 | 31.80 |
|  |  |  | 3 | 0.1 | 8.69 | 34.1 | 0.344 | 25.50 |  | 1.88 | 14.5 | 0.051 | 31.70 |

NO_3_^-^, nitrate; NH_4_^+^, ammonium; N, nitrogen; P, phosphorus; NA, insufficient material available to conduct chemical analyses.

^†^ Replicate used in preliminary assessment using forest floors and mineral soils collected in 2013 and 2014.

**Table S2:** Percent inertia associated with reclamation status or potentially confounding factors in constrained ordinations of the relative abundance of fungal taxa and functional groups, the presence/absence of fungal taxa, and the number of taxa belonging to functional groups in preliminary assessment of mineral soils and/or forest floors.

|  |  |  |  |  |  |  | **PCNM** |  |
| --- | --- | --- | --- | --- | --- | --- | --- | --- |
| **Response** | **Soil layer** | **Reclamation status** | **Tree species** | **Regeneration Year** | **Stand stage** | **Tree status** | **Model** | **Components** |
| **Fungal taxa** | |  |  |  |  |  |  |  |
| Relative abundance | |  |  |  |  |  |  |  |
|  | Mineral soils | 16.20** | 23.25 | 8.48 | 19.24 | 6.74 | 64.07*** | 1, 2, 4, 5, 6 |
|  | Forest floors | 15.19** | 21.06 | 11.90 | 17.56 | 9.09 | 27.41*** | 1, 2 |
|  | Both soil layers ^†^ | 10.14*** | 13.57** | 6.21 | 10.66 | 4.54 | 31.06*** | 1, 2, 5, 6 |
| Presence / absence | |  |  |  |  |  |  |  |
|  | Mineral soils | 21.64** | 21.93 | 7.72 | 23.08 | 12.52 | 21.51** | 1 |
|  | Forest floors | 15.63** | 24.62* | 8.42 | 16.93 | 8.72 | 27.14** | 1, 6 |
|  | Both soil layers ^†^ | 14.05*** | 15.40*** | 5.04 | 13.27** | 7.16* | 38.59*** | 1, 2, 3, 5, 6 |
| **Functional groups** | |  |  |  |  |  |  |  |
| Relative abundance | |  |  |  |  |  |  |  |
|  | Mineral soils | 20.80*** | 24.29 | 6.96 | 18.83 | 10.21 | 21.55*** | 1 |
|  | Forest floors | 12.34 | 28.53 | 4.38 | 10.23 | 5.08 | ns |  |
|  | Both soil layers ^†^ | 8.30* | 17.32* | 3.53 | 10.20 | 4.99 | 26.36*** | 1, 3, 6 |
| Number of taxa | |  |  |  |  |  |  |  |
|  | Mineral soils | 36.15** | 22.35 | 4.09 | 17.09 | 9.02 | 36.02** | 1 |
|  | Forest floors | 29.29** | 36.43 | 5.20 | 18.44 | 11.71 | 49.04*** | 1, 5 |
|  | Both soil layers ^†^ | 23.99*** | 17.18* | 2.84 | 12.42 | 7.03 | 31.10*** | 1, 5 |

PCNM, principal coordinates of neighbour matrices; ns, non-significant.

^†^ Models were each conditioned by soil fraction.**Table S3**: Variance partitioning (%) between reclamation status and other significant confounding factors for fungal taxa (relative abundance and presence/absence) or functional groups (relative abundance and number of taxa belonging to each group) in preliminary assessment of jack pine, white spruce and Siberian larch soils in the forestry and reclamation sites. Community data from mineral soils and forest floors were treated separately and/or jointly.

| **Response** | **Soil layer** | **Reclamation status** | **Shared** | **Other factors** | **Residual** |
| --- | --- | --- | --- | --- | --- |
| **Fungal taxa** | |  |  |  |  |
| Relative abundance | |  |  |  |  |
|  | Mineral soils | 16.20* | 0 | 48.02 | 35.78 |
|  | Forest floors | 15.17** | 0.02 | 12.29 | 72.52 |
|  | Both soil layers ^†^ | 6.86** | 3.27 | 32.42 | 57.44 |
| Presence/absence | |  |  |  |  |
|  | Mineral soils | 21.64** | 0 | 0 | 78.36 |
|  | Forest floors | 13.59 | 2.04 | 33.89 | 50.48 |
|  | Both soil layers ^†^ | 6.47** | 7.59 | 45.90 | 40.05 |
| **Functional groups** | |  |  |  |  |
| Relative abundance | |  |  |  |  |
|  | Mineral soils | 20.80** | 0 | 0 | 79.20 |
|  | Forest floors | 0 | 0 | 0 | 0 |
|  | Both soil layers ^†^ | 6.40 | 1.90 | 28.63 | 63.07 |
| Number of taxa | |  |  |  |  |
|  | Mineral soils | 36.15*** | 0 | 0 | 63.85 |
|  | Forest floors | 29.29** | 0 | 19.76 | 50.95 |
|  | Both soil layers ^†^ | 14.14*** | 9.84 | 13.88 | 62.14 |

* Significant reclamation status effects at the 0.05 probability level

** Significant reclamation status effects at the 0.01 probability level

*** Significant reclamation status effects at the 0.001 probability level

^†^ Models were each conditioned by soil fraction.

**Table S4:** Preliminary assessment of fungal taxa consistently associated with either reclamation or forestry sites when considering both the relative abundance and presence/absence of taxa in mineral soils and the relative abundance of taxa in forest floors.

| **Reclamation sites** | |  |  | **Forestry sites** | | |  |
| --- | --- | --- | --- | --- | --- | --- | --- |
| **Total read number** | **Trophic function**^†^ | **Fungal taxon** |  | **Total read number** | **Trophic function**^†^ | **Fungal taxon** | |
| 1791 | EM | *Tomentella* sp. LM5498 |  | 10,118 | ST | *Geminibasidium hirsutum* | |
| 1042 | ST | *Infundichalara microchona* |  | 7542 | UK | *Calyptrozyma arxii* | |
| 891 | EM | *Tomentella* sp. OTU204 |  | 3918 | EM | *Amphinema* sp. 2 UK-2011 | |
| 782 | ST | *Mortierella* sp. FSU 10731 |  | 1638 | ST | Trechisporales | |
| 724 | UK | *Knufia peltigerae* |  | 1179 | ST | *Fayodia gracilipes* | |
| 620 | EM | *Hebeloma* sp. LM4904 |  | 773 | ST | *Hyalodendriella betulae* | |
| 607 | EM | *Tomentella* sp. OTU199 |  | 763 | UK | fungal sp. JH 109 | |
| 514 | ST | *Hemimycena ochrogaleata* |  | 577 | ST | Dothideomycetes sp. genotype 65 | |
| 498 | UK | fungal sp. Sen013 |  | 466 | EM | Atheliaceae sp. H353-2 | |
| 361 | ST | *Lycoperdon* cf. *niveum* MJ4068 |  | 373 | ST | Herpotrichiellaceae sp. RB-2011 | |
| 352 | AP | *Beauveria bassiana* |  | 370 | UK | *Ambomucor seriatoinflatus* var. XYL-2013a | |
| 274 | PP | *Phoma herbarum* |  | 367 | EM | *Tomentella* sp. LT56 | |
| 273 | ST | *Cladosporium* sp. 0511MAR12L2 |  | 350 | EM | *Piloderma sphaerosporum* | |
| 267 | SWR | *Hyphodontia alutaria* |  | 269 | ST | *Coniochaeta* sp. XS62m1 | |
| 262 | ST | *Myrothecium* sp. REF176 |  | 257 | ST | *Umbelopsis isabellina* | |
| 245 | ST | *Mycena tenax* |  | 221 | ST | Basidiomycota sp. CC 02-22 | |
| 241 | SY | *Leucosporidium fasciculatum* |  | 194 | ST | *Mycena adonis* var. *adonis* | |
| 211 | ST | *Stromatonectria caraganae* |  | 140 | EM | Sebacinales sp. PC38 | |
| 188 | EM | *Inocybe malenconii* |  | 125 | SBR | *Rhizoctonia* sp. 251 | |
| 168 | ST | foliar endophyte of *Picea glauca* sp. K2 |  | 117 | ST | *Ramariopsis flavescens* | |
| 155 | ST | Hypocreales sp. GPO_CO_01_H6 |  | 114 | UK | Ascomycota sp. AR-2010 | |
| 154 | UK | Ascomycota sp. BDJ |  | 89 | UK | fungal sp. mh4293.4 | |
| 134 | ST | Helotiales sp. Rr136 |  | 67 | EM | *Tomentellopsis* sp. BB-2010 | |
| 131 | ST | *Mortierella* sp. nwa_ca_0101g |  | 60 | UK | fungal sp. JH 144 | |
| 125 | ST | *Xenopolyscytalum* sp. CCF 4159 |  | 54 | MP | *Rhodosporidium concentricum* | |
| 119 | UK | melanized limestone ascomycete CR-2004 |  | 37 | ST | *Hyaloscypha aureliella* | |
| 104 | ST | *Mortierella sclerotiella* |  | 29 | ST | *Umbelopsis ramanniana* | |
| 98 | ST | Helotiales sp. ARON3038.S |  | 26 | UK | fungal sp. ARIZ AZ0501 | |
| 91 | ST | Pleosporales sp. 29_83F |  | 25 | EM | *Rhizopogon evadens* | |
| 91 | UK | fungal sp. acwVHB5_18 |  | 22 | ST | *Phialocephala virens* | |
| 83 | ST | *Thelebolus* sp. CBS 137501 |  | 21 | EM | *Inocybe splendentoides* | |
| 82 | ST | *Mortierella* sp. TUFC 20049 |  | 19 | EM | *Thelephora* aff. *terrestris* H5C_2 | |
| 79 | UK | fungal sp. CtFE 32 |  | 19 | EM | *Thelephora terrestris* | |
| 73 | ST | *Mortierella* sp. UASWS0878 |  | 18 | SP | *Pseudogymnoascus roseus* | |
| 70 | ST | zygomycete sp. olrim272 |  | 16 | EM | *Russula fragilis* | |
| 57 | SY | *Leucosporidiella* sp. BEA-2010 |  | 15 | EM | *Sebacina* sp. Seb11I | |
| 56 | EM | *Inocybe amethystina* |  | 13 | ST | Helotiales sp. C20 | |
| 51 | ST | *Helicodendron luteoalbum* |  | 12 | EM | *Athelia* sp. HHB-15599 | |
| 51 | EM | Sebacinales sp. JPK 89 |  |  |  |  | |
| 49 | ST | *Phialea strobilina* |  |  |  |  | |
| 49 | UK | fungal sp. Cen003 |  |  |  |  | |
| 49 | UK | fungal sp. NLR-2013 |  |  |  |  | |
| 43 | ST | *Stilbella* sp. 1 TMS-2011 |  |  |  |  | |
| 41 | ST | *Podospora glutinans* |  |  |  |  | |
| 38 | UK | *Paraphoma chrysanthemicola* |  |  |  |  | |
| 36 | ST | Dothideomycetes sp. DC2611 |  |  |  |  | |
| 35 | SBR | *Ceratobasidium* sp. MB-2014b |  |  |  |  | |
| 34 | ST | basidiomycete sp. RT00050 |  |  |  |  | |
| 31 | L | *Degelia plumbea* |  |  |  |  | |
| 30 | ST | Basidiomycota cf. Tremellales OTU_033 |  |  |  |  | |
| 30 | UK | fungal sp. D5-(2)II2 |  |  |  |  | |
| 23 | PP | *Microdochium nivale* |  |  |  |  | |
| 22 | PP | *Verticillium leptobactrum* |  |  |  |  | |
| 17 | ST | Basidiomycota sp. CC 15-08 |  |  |  |  | |
| 17 | UK | *Knufia petricola* |  |  |  |  | |
| 15 | ST | leaf litter ascomycete strain its414 |  |  |  |  | |
| 14 | ST | *Clitocybe phyllophila* |  |  |  |  | |
| 14 | ST | *Dothideomycetes* sp. gtype 934 JMUR-2014 |  |  |  |  | |
| 13 | ST | Basidiomycota sp. CBS 100.26 |  |  |  |  | |
| 13 | ST | *Mucor* sp. FL-2014 |  |  |  |  | |
| 12 | ST | *Anthostomella conorum* |  |  |  |  | |
| 12 | ST | *Chalara* sp. OTU_106 |  |  |  |  | |
| 12 | EM | Thelephoraceae sp. Taylor #2 |  |  |  |  | |

Partially constrained models accounted for respective significant confounding factors. Taxa are ordered by total read number across all samples. Only taxa each representing more than 0.01% of total reads are listed.

^†^ UK, unknown function or fungal taxa not identified; EM, ectomycorrhizal; AM, arbuscular mycorrhizal; PP, plant pathogen; ST, saprotroph; SY, saprotroph yeast; SFY; saprotroph facultative yeast; SBR, saprotroph brown rot; L, lichenized; SWR, saprotroph white rot; AP, animal parasite; ER; ericoid; MP, mycoparasite; EP, endophyte; SP, saprotroph pathogen

**Table S5:** Percent inertia associated with reclamation status and potentially confounding factors in constrained ordinations (constrained correspondence analyses) of the relative abundance and presence/absence of fungal taxa in coarse soils, fine soils, forest floors and/or roots in jack pine transects.

|  |  |  |  |  | **Nutrients** |  |  | **PCNM** |  |
| --- | --- | --- | --- | --- | --- | --- | --- | --- | --- |
| **Response** | **Soil fraction** | **Reclamation status** | **Regen. year** | **Forest floor depth** | **Model** | **Variables** |  | **Model** | **Components** |
| Relative abundance | |  |  |  |  |  |  |  |  |
|  | Coarse soils ^†^ | 18.32** | 6.86 | 14.50** | 29.04*** | NO_3_, P |  | 17.99*** | 1 |
|  | Coarse soils ^‡^ | 17.79** | 8.67 | 14.89* | 30.43*** | NO_3_, P |  | 17.75** | 1 |
|  | Fine soils ^‡^ | 18.65** | 9.91 | 12.05 | 32.79** | NO_3_, P |  | 19.00*** | 1 |
|  | Roots ^‡^ | 13.22** | 9.89 | 11.45* | 13.13** | P |  | 13.14*** | 1 |
|  | Forest floors ^‡^ | 16.88** | 15.64** | 12.06 | 16.74** | P |  | 16.57** | 1 |
|  | Both roots and coarse soils ^‡,§^ | 10.82*** | 6.25* | 8.98*** | 17.50*** | NO_3_, P |  | 17.27*** | 1, 2 |
|  | All soil fractions ^‡,§^ | 7.75*** | 3.46* | 5.48*** | 15.72*** | NO_3_, NH_4_, P |  | 11.72*** | 1, 2 |
| Presence/absence | |  |  |  |  |  |  |  |  |
|  | Coarse soils ^†^ | 28.65** | 6.61 | 24.06*** | 27.93** | P |  | 26.72** | 1 |
|  | Coarse soils ^‡^ | 21.94** | 9.43 | 20.34*** | 21.87** | P |  | 21.31** | 1 |
|  | Fine soils ^‡^ | 26.37** | 12.55 | 19.56** | 40.48*** | NO_3_, P |  | 25.05** | 1 |
|  | Roots ^‡^ | 16.66** | 9.05 | 13.40** | 26.95*** | NO_3_, P |  | 16.28** | 1 |
|  | Forest floors ^‡^ | 19.58** | 16.80* | 15.90* | 19.36** | P |  | 32.53*** | 1, 2 |
|  | Both roots and coarse soils ^‡,§^ | 15.39*** | 6.40 | 12.23*** | 28.82*** | NO_3_, NH_4_, P |  | 22.24*** | 1, 2 |
|  | All soil fractions ^‡,§^ | 13.53*** | 4.70** | 10.06*** | 27.11*** | NO_3_, NH_4_, Total N, P |  | 18.69*** | 1, 2 |

PCNM, principal coordinates of neighbour matrices.

* Significant reclamation status effects at the 0.05 probability level

** Significant reclamation status effects at the 0.01 probability level

*** Significant reclamation status effects at the 0.001 probability level

^†^ Communities sequenced using Illumina sequencing.

^‡^ Communities sequenced using pyrosequencing.

^§^ Models were each conditioned by soil fraction.

**Table S6:** Fungal taxa detected by pyrosequencing that were consistently associated (in terms of both relative abundance and presence/absence) with root and coarse soil samples from either reclamation or forestry jack pine transects. *

| **Reclamation transects** | | |  | | |  | **Forestry transects** | | |  | |
| --- | --- | --- | --- | --- | --- | --- | --- | --- | --- | --- | --- |
| **Total read number** | **Trophic function**^†^ | **Fungal taxon** | |  | **Total read number** | | | **Trophic function**^†^ | **Fungal taxon** | |  |
| 3883 | EM | *Tricholoma aurantium* ^‡^ | |  | 11,450 | | | ST | *Geminibasidium hirsutum* ^§^ | |  |
| 2141 | EM | *Tomentella* sp. OTU204 ^§^ | |  | 9155 | | | UK | *Calyptrozyma arxii* ^§^ | |  |
| 2017 | EM | *Inocybe splendens* var. *phaeoleuca* | |  | 4226 | | | EM | *Russula decolorans* | |  |
| 1983 | EM | *Tomentella badia* | |  | 2219 | | | EM | *Cenococcum geophilum* | |  |
| 1374 | ST | *Infundichalara microchona* ^§^ | |  | 1677 | | | ST | Basidiomycota sp. CC 02-22 ^§^ | |  |
| 1176 | ST | *Microscypha ellisii* ^§^ | |  | 1316 | | | ST | *Mortierella* sp. 1 JAS-2013 | |  |
| 1057 | ST | *Mortierella* sp. FSU 10731 ^§^ | |  | 1010 | | | EM | Sebacinaceae sp. A (JLP 995) | |  |
| 1013 | UK | fungal sp. Sen013 ^§^ | |  | 874 | | | ST | Trechisporales ^§^ | |  |
| 885 | EM | *Inocybe amethystine* ^§^ | |  | 777 | | | ST | Dothideomycetes sp. genotype 65 ^§^ | |  |
| 729 | AP | *Beauveria bassiana* ^§^ | |  | 738 | | | EM | *Russula* sp. N21 | |  |
| 538 | UK | fungal sp. CtFE 32 ^§^ | |  | 654 | | | EM | *Piloderma sphaerosporum* ^§^ | |  |
| 536 | ST | Helotiales sp. ARON3038.S ^§^ | |  | 649 | | | ST | *Helicoon* aff. *maioricense* ICMP14920 | |  |
| 437 | PP | *Leptosphaeria sclerotioides* | |  | 471 | | | EM | *Tomentella* sp. LT56 ^§^ | |  |
| 409 | PP | *Clonostachys* sp. G435 | |  | 427 | | | ST | *Clitopilus hirneolus* | |  |
| 303 | SWR | *Leucoagaricus sericifer* | |  | 393 | | | ST | *Mycena adonis* var. *adonis* ^§^ | |  |
| 244 | ST | *Stromatonectria caraganae* ^§^ | |  | 337 | | | UK | fungal sp. acwVHT30_4 ^‡^ | |  |
| 223 | ER | *Oidiodendron* sp. KO-groupD 2014 ^‡^ | |  | 322 | | | ST | *Clitopilopsis hirneola* | |  |
| 221 | SBR | *Ceratobasidium* sp. MB-2014b ^§^ | |  | 295 | | | EM | Sebacinales sp. JPK 89 ^‡^ | |  |
| 215 | UK | *Knufia peltigerae* ^§^ | |  | 204 | | | UK | fungal sp. 1 RB-2011 | |  |
| 209 | PP | *Collophora* sp. OTU_016 | |  | 156 | | | ST | *Stilbella* sp. 1 TMS-2011 ^‡^ | |  |
| 176 | UK | fungal sp. 9DI1-7B | |  | 140 | | | UK | Ascomycota sp. AR-2010 ^§^ | |  |
| 176 | UK | fungal sp. acwVHB5_18 ^§^ | |  | 132 | | | ST | Helotiaceae sp. II GK-2010 | |  |
| 145 | ST | Helotiales sp. GPO_CO_01_G12 | |  | 113 | | | MP | *Rhodosporidium concentricum* ^§^ | |  |
| 140 | UK | *Pseudorobillarda phragmitis* | |  | 104 | | | UK | fungal sp. mh4293.4 ^§^ | |  |
| 132 | ST | *Hemimycena gracilis* | |  | 93 | | | EM | *Calluna vulgaris* root associated fungus | |  |
| 130 | ST | *Strumella griseola* | |  | 89 | | | ST | Mortierellaceae sp. SN-2008 ^‡^ | |  |
| 124 | ST | *Hemimycena ochrogaleata* ^§^ | |  | 85 | | | UK | fungal sp. JH 144 ^§^ | |  |
| 124 | UK | melanized limestone ascomycete CR-2004 ^§^ | |  | 82 | | | EM | *Thelephora* aff. *terrestris* H5C_2 ^§^ | |  |
| 106 | ST | *Mortierella sclerotiella* ^§^ | |  | 76 | | | ST | fungal endophyte sp. O8-3161 | |  |
| 103 | ST | *Pseudeurotium bakeri* | |  | 72 | | | UK | fungal sp. KRP11 ^‡^ | |  |
| 102 | SWR | *Hyphodontia alutaria* ^§^ | |  | 72 | | | EM | *Tomentellopsis* sp. BB-2010 ^§^ | |  |
| 99 | UK | fungal sp. Cen003 ^§^ | |  | 70 | | | PP | *Fusarium* sp. HS6 ^‡^ | |  |
| 86 | UK | *Thyronectria caudata* ^§^ | |  | 61 | | | ST | *Hyaloscypha aureliella* ^§^ | |  |
| 83 | ST | *Mortierella* sp. TUFC 20049 ^§^ | |  | 61 | | | ST | *Umbelopsis isabellina* ^§^ | |  |
| 69 | UK | fungal sp. V-T2 | |  | 49 | | | L | Lecanoromycetidae sp. RB-2011 | |  |
| 68 | ST | Helotiales sp. ARON3063 | |  | 49 | | | ST | *Massarina* sp. MUT 4323 ^‡^ | |  |
| 62 | EM | *Wilcoxina* sp. aurim720 | |  | 45 | | | ST | *Phaeosphaeria* sp. OTU_014 ^‡^ | |  |
| 61 | ST | *Cladosporium herbarum* | |  | 45 | | | ST | *Umbelopsis ramanniana* ^§^ | |  |
| 60 | UK | fungal sp. NLR-2013 ^§^ | |  | 43 | | | ST | Helotiales sp. DU60 | |  |
| 58 | EM | *Inocybe splendentoides* | |  | 40 | | | EM | *Inocybe malenconii* ^‡^ | |  |
| 55 | ST | *Sporormiella vexans* ^‡^ | |  | 37 | | | UK | fungal sp. ARIZ AZ0501 ^§^ | |  |
| 53 | ST | *Articulospora proliferata* | |  | 37 | | | ST | Herpotrichiellaceae sp. RB-2011 ^§^ | |  |
| 53 | ST | *Phialea strobilina* ^§^ | |  | 34 | | | EM | *Pseudotomentella larsenii* | |  |
| 50 | SY | *Fellomyces* sp. CBS 8304; 8276; 8309 | |  | 33 | | | UK | fungal sp. acwVHT5_8 ^‡^ | |  |
| 47 | ST | Basidiomycota sp. CBS 100.26 ^§^ | |  | 30 | | | UK | Ascomycota sp. BBC | |  |
| 44 | UK | fungal sp. KRP96 | |  | 26 | | | SP | *Pseudogymnoascus roseus* ^§^ | |  |
| 44 | PP | *Olpidium brassicae* | |  | 23 | | | ST | Dothideomycetes sp. genotype 934 JMUR-2014 ^‡^ | |  |
| 43 | SFY | *Cladophialophora* sp. FMR 10591 | |  | 22 | | | ST | Helotiales sp. 13 CG-2012 ^§^ | |  |
| 40 | ST | *Acremonium nepalense* | |  | 22 | | | L | *Placynthiella icmalea* | |  |
| 40 | UK | fungal sp. D2 | |  | 20 | | | ST | *Lachnellula pulverulenta* | |  |
| 39 | ST | foliar endophyte of *Picea glauca* sp. K2 ^§^ | |  | 20 | | | ST | *Myrothecium* sp. Sn342 ^‡^ | |  |
| 38 | EM | *Lyophyllum* cf. *rhopalopodium* AB99-10-232 ^§^ | |  | 19 | | | SY | *Kockovaella machilophila* | |  |
| 37 | PP | *Plectosphaerella cucumerina* | |  | 18 | | | ST | *Thelebolus* sp. CBS 137501 ^‡^ | |  |
| 33 | PP | *Gaeumannomyces graminis* var. *tritici* | |  |  | | |  |  | |  |
| 31 | UK | fungal sp. D5-(2)II2 ^§^ | |  |  | | |  |  | |  |
| 30 | UK | fungal sp. ANTELF33 | |  |  | | |  |  | |  |
| 29 | ST | *Lycoperdon* cf. *niveum* MJ4068 ^§^ | |  |  | | |  |  | |  |
| 22 | SY | *Cryptococcus podzolicus* | |  |  | | |  |  | |  |
| 20 | PP | *Nectria berolinensis* | |  |  | | |  |  | |  |
| 19 | UK | ascomycete sp. IZ-1932 | |  |  | | |  |  | |  |
| 19 | EM | *Tomentella* sp. OTU221 ^§^ | |  |  | | |  |  | |  |
| 17 | EM | *Sebacina* sp. Rr35 | |  |  | | |  |  | |  |

* Data were analyzed in constrained ordination models conditioned by respective significant confounding factors. Taxa are ordered by total read number across all samples. Only taxa each representing more than 0.01% of total reads are listed. ^†^ UK, unknown function or fungal taxon not identified; EM, ectomycorrhizal; AM, arbuscular mycorrhizal; PP, plant pathogen; ST, saprotroph; SY, saprotroph yeast; SFY; saprotroph facultative yeast; SBR, saprotroph brown rot; L, lichenized; SWR, saprotroph white rot; AP, animal parasite; ER; ericoid; MP, mycoparasite; EP, endophyte; SP, saprotroph pathogen. ^‡^ Taxa not significantly associated with area in models not conditioned by confounding factors. ^§^ Taxa also significantly associated with area in preliminary assessment using 2013-2014 samples.

**Table S7:** Percent inertia associated with reclamation status and potentially confounding factors in constrained ordinations (redundancy analyses) of the relative abundances of functional groups and the number of fungal taxa belonging to functional groups in coarse soils, fine soils, forest floors, and/or roots in jack pine transects.

|  |  | |  |  |  | **Nutrients** |  |  | **PCNM** |  |
| --- | --- | --- | --- | --- | --- | --- | --- | --- | --- | --- |
| **Response** | **Soil fraction** | | **Reclamation status** | **Regen. year** | **Forest floor depth** | **Model** | **Variables** |  | **Model** | **Components** |
| Relative abundance | |  | |  |  |  |  |  |  |  |
|  | Coarse soils ^†^ | | 17.95* | 8.49 | 20.05* | 38.95** | NH_4_, P |  | 38.80** | 1, 2 |
|  | Coarse soils ^‡^ | | 29.73** | 9.22 | 26.28** | 49.07*** | NO_3_, P |  | 31.70*** | 1 |
|  | Fine soils ^‡^ | | 24.42** | 9.45 | 15.78 | 44.59** | NH_4_, P |  | 42.63** | 1, 2 |
|  | Roots ^‡^ | | 12.07 | 14.27* | 9.40 | ns |  |  | ns |  |
|  | Forest floors ^‡^ | | 11.90 | 10.77 | 25.61** | ns |  |  | 19.36* | 2 |
|  | Both roots and coarse soils ^‡,§^ | | 14.18*** | 8.29* | 8.17* | 14.83*** | P |  | 23.00*** | 1, 2 |
|  | All soil fractions ^‡,§^ | | 10.20*** | 2.42 | 7.25*** | 18.02*** | NO_3_, P |  | 17.33*** | 1, 2 |
| Number of taxa | |  | |  |  |  |  |  |  |  |
|  | Coarse soils ^†^ | | 37.13** | 4.18 | 36.24** | 37.62* | Total N |  | 30.87* | 1 |
|  | Coarse soils ^‡^ | | 46.46** | 5.61 | 37.88** | 45.92** | P |  | 43.61** | 1 |
|  | Fine soils ^‡^ | | 35.44** | 9.62 | 22.68* | 51.74*** | NH_4_, P |  | 33.66** | 1 |
|  | Roots ^‡^ | | 30.91** | 8.50 | 21.59** | 29.83** | P |  | 27.71** | 1 |
|  | Forest floors ^‡^ | | 28.51* | 10.83 | 28.68* | 32.89** | NO_3_ |  | ns |  |
|  | Both roots and coarse soils ^‡,§^ | | 32.98*** | 4.81 | 22.79*** | 40.83*** | NH_4_, P |  | 38.21*** | 1, 2 |
|  | All soil fractions ^‡,§^ | | 26.26*** | 3.50 | 18.77*** | 33.41*** | NH_4_, P |  | 30.49*** | 1, 2 |

PCNM, principal coordinates of neighbour matrices; Percentages are omitted for nonsignificant (ns) models constrained by nutrients or principal coordinates of neighbour matrices (PCNM).

* Significant reclamation status effects at the 0.05 probability level

** Significant reclamation status effects at the 0.01 probability level

**Table S8**: Fungal taxa consistently detected by Illumina sequencing in all coarse soil samples in jack pine transects.

| **Total read number** | **Trophic function**^†^ | **Fungal taxa** |
| --- | --- | --- |
| 558 | EM | *Tricholoma matsutake* clone J28 |
| 17,867 | EM | *Wilcoxina rehmii* isolate T3PK24 |
| 25,198 | SP | *Pseudogymnoascus pannorum* strain MKOTU5 |
| 210 | PP | *Epicoccum nigrum* isolate KK7.1 |
| 2734 | PP | *Phoma* *herbarum* isolate JM_TPBP033 |
| 277 | ST | *Cladosporium* sp. 18S ribosomal RNA gene |
| 7301 | ST | *Mortierella alpina* isolate KK9 |
| 1509 | ST | *Mortierella alpina* strain FSU 2698 |
| 6226 | ST | *Mortierella alpina* strain MKOTU34 |
| 19,578 | ST | *Mortierella* sp. FSU 10731 |
| 1241 | ST | *Penicillium chalabudae* CBS 219.66 |
| 660 | ST | *Penicillium spinulosum* strain SmDk8 |
| 543 | UK | *Patinella hyalophaea* voucher H.B. 9739 |
| 662 | SFY | *Cladophialophora chaetospira* |

^†^ EM, ectomycorrhizal; PP, plant pathogen; SFY; saprotroph facultative yeast; SP, saprotroph pathogen; ST, saprotroph; UK, unknown function.

**Table S9:** Fungal taxa detected by Illumina sequencing consistently associated (in terms of both relative abundance and presence/absence) with samples from either reclamation or forestry jack pine transects. *

| **Reclamation transects** | | |  | | |  | **Forestry transects** | |  | |
| --- | --- | --- | --- | --- | --- | --- | --- | --- | --- | --- |
| **Total read number** | **Trophic function**^†^ | **Fungal taxon** | |  | **Total read number** | | **Trophic function**^†^ | **Fungal taxon** | |  |
| 4945 | EM | *Lyophyllum decastes* voucher LAS06-152 | |  | 31,611 | | ST | *Geminibasidium hirsutum* strain DAOM 2419 | |  |
| 3546 | EM | *Tomentella* sp. voucher MES-2144 | |  | 21,599 | | ST | *Mortierella* sp. TR158 | |  |
| 2903 | noID | OTU_75 | |  | 17,439 | | EM | *Russula* sp. GAL15390 | |  |
| 2523 | noID | OTU_90 | |  | 17,433 | | EM | *Russula decolorans* voucher SMI265 | |  |
| 2348 | ST | *Mortierella gamsii* strain E2A(5) | |  | 15,304 | | ST | *Sagenomella humicola* | |  |
| 2230 | noID | OTU_92 | |  | 11,069 | | EM | *Piloderma sphaerosporum* isolate 180 | |  |
| 2189 | ST | *Chalara hyalocuspica* isolate 385Ja14 | |  | 9526 | | UK | *Calyptrozyma arxii* CBS 354.92 | |  |
| 2165 | ST | *Cadophora* sp. strain F1B(7B) | |  | 4923 | | EM | *Tylospora* sp. 1 LR-2015 voucher UC202292 | |  |
| 1763 | PP | *Verticillium* sp. EXP0564F | |  | 4870 | | ST | *Paratritirachium curvibasidium* strain DA | |  |
| 1701 | ST | *Cadophora* sp. voucher M Loden | |  | 4735 | | EM | *Tricholoma luteomaculosum* voucher UBC F1 | |  |
| 1664 | EM | *Sebacina vermifera* isolate FFP337 | |  | 4446 | | EM | *Russula persicina* 499RUS26 | |  |
| 1660 | noID | OTU_117 | |  | 3906 | | ST | *Luellia recondita* isolate 217 | |  |
| 1540 | noID | OTU_105 | |  | 3168 | | EM | *Thelephora terrestris* isolate O4Wr_4 | |  |
| 1382 | ST | *Tricharina ascophanoides* | |  | 2875 | | ST | *Phialocephala* sp. clone PM69AR | |  |
| 1347 | UK | *Trichothecium crotocinigenum* strain D2A | |  | 2621 | | noID | *Epacris microphylla* root associated fungi 1 | |  |
| 1325 | ST | *Schizothecium glutinans* strain CBS 134.8 | |  | 2210 | | EM | *Tomentellopsis* sp. isolate MT38 | |  |
| 1141 | ST | *Acremonium* sp. LB-2012 | |  | 1808 | | noID | Salal root associated fungus UBCtra143 | |  |
| 1073 | EM | *Helvellosebacina* sp. TUB 020028 | |  | 1615 | | noID | Venturiaceae sp. GM 15-02 | |  |
| 1034 | ST | *Mortierella* sp. ATT235 | |  | 1483 | | EM | *Tomentella* sp. &apos;LT56&apos | |  |
| 977 | ST | *Eucasphaeria capensis* isolate 2712 | |  | 1407 | | ER | *Rhizoscyphus ericae* isolate FFP629 | |  |
| 856 | PP | *Olpidium virulentus* isolate CTL-1 | |  | 1245 | | EM | *Suillus glandulosipes* isolate CO4 | |  |
| 850 | EM | *Tomentella* sp. voucher Ch2 | |  | 1058 | | EM | *Sebacina* sp. Seb13I | |  |
| 834 | noID | *Metapochonia suchlasporia* voucher CFMR:F | |  | 992 | | EM | *Hydnellum peckii* voucher SL57 | |  |
| 726 | ST | *Mortierella gamsii* strain S6A1CV | |  | 904 | | ST | *Gelasinospora* sp. VL222 | |  |
| 715 | UK | *Knufia* sp. strain F1A(3) | |  | 814 | | ST | *Coniochaeta mutabilis* isolate 210Ja14 | |  |
| 706 | ST | *Stilbella* sp. 1 TMS-2011 voucher SC12d50 | |  | 744 | | ST | *Mortierella minutissima* strain FSU 2735 | |  |
| 683 | SFY | *Exophiala equina* strain FMR 10881 | |  | 630 | | ST | *Geminibasidium donsium* strain DAOM 24196 | |  |
| 599 | SFY | *Phialophora* sp. DF33 | |  | 617 | | MP | *Rhodosporidium concentricum* isolate 9908 | |  |
| 586 | ST | *Cadophora* sp. Di3-8 | |  | 616 | | ST | *Mortierella verticillata* isolate 90Jb14 | |  |
| 563 | AP | *Beauveria bassiana* isolate KJ4 | |  | 552 | | EM | *Tricholoma saponaceum* var. *saponaceum* | |  |
| 555 | MP | *Cosmospora viridescens* strain CBS 102430 | |  | 529 | | ER | *Oidiodendron chlamydosporicum* UAMH 6520 | |  |
| 551 | ST | *Lasiosphaeria glabrata* strain SMH4617 | |  | 439 | | EM | *Phellodon melaleucus* voucher E00219373 | |  |
| 514 | SP | *Pseudogymnoascus pannorum* strain MKOTU20 | |  | 426 | | EM | *Inocybe lacera* | |  |
| 491 | noID | OTU_216 | |  | 416 | | noID | OTU_244 | |  |
| 482 | ST | *Phialocephala virens* CBS 452.92 | |  | 394 | | EM | *Russula exalbicans* voucher IK-00547 ^‡^ | |  |
| 422 | noID | OTU_229 | |  | 348 | | noID | *Epacris microphylla* root associated fungi 2 | |  |
| 403 | ST | *Oliveonia pauxilla* voucher KC1068 | |  | 311 | | ST | *Xenochalara* sp. sd1cN3c | |  |
| 391 | noID | OTU_227 | |  | 287 | | EM | *Russula decolorans* isolate OUC99188 | |  |
| 384 | UK | *Thyronectria concentrica* strain ALLM | |  | 240 | | ST | *Basidiodendron caesiocinereum* voucher UC | |  |
| 369 | EP | *Cystodendron* sp. EXP0561F | |  | 229 | | PP | *Phoma herbarum* isolate OK-RAW-2 | |  |
| 341 | noID | Ericoid mycorrhizal sp. Sm8 | |  | 228 | | ST | *Hygrocybe conica* isolate DD2 | |  |
| 320 | noID | OTU_304 | |  | 210 | | noID | OTU_395 | |  |
| 310 | EM | *Inocybe amethystina* voucher 5055 | |  | 210 | | EM | *Amphinema* sp. MB-2011 | |  |
| 291 | SFY | *Phialophora* sp. C16 isolate C16 | |  | 199 | | ST | *Clavulinopsis* sp. voucher MES-1617 ^‡^ | |  |
| 283 | EM | *Tomentella coerulea* isolate PA46 | |  | 189 | | ST | *Scytinostroma* sp. DLL2011-1 voucher CFMR | |  |
| 281 | noID | OTU_274 | |  | 182 | | ST | *Sydowia polyspora* isolate 361Jc14 | |  |
| 242 | noID | OTU_361 | |  | 179 | | ST | *Claussenomyces* sp. PDD 55517 | |  |
| 231 | MP | *Hypocrea lixii* strain JB T1244 | |  | 178 | | EM | *Piloderma sphaerosporum* isolate FFP835 | |  |
| 203 | noID | Sporormiaceae sp. isolate L32 | |  | 176 | | SFY | *Cladophialophora* sp. L3-3-NN-2016 | |  |
| 196 | MP | *Syncephalis* sp. KL-2016a clone S526-M05- | |  | 151 | | ST | *Umbelopsis angularis* CBS 603.68 | |  |
| 190 | EM | *Inocybe flocculosa* strain HRL1870 | |  | 149 | | EM | *Inocybe purpureobadia* isolate K217 | |  |
| 185 | EM | *Tomentella ellisii* clone NS208 | |  | 147 | | EM | *Lyophyllum aemiliae* AB04-10-467 isolate | |  |
| 180 | noID | OTU_360 | |  | 143 | | EM | *Tomentellopsis submollis* isolate FFP892 | |  |
| 177 | noID | *Chlamydocillium cyanophilum* isolate CBS5 | |  | 143 | | ST | *Coniochaeta navarrae* strain LTA3 | |  |
| 172 | noID | OTU_389 | |  | 141 | | EM | *Thelephora terrestris* voucher K15 | |  |
| 169 | noID | *Parathyridaria ramulicola* CBS 141479 | |  | 138 | | noID | Lecanoromycetidae sp. RB-2011 voucher LF | |  |
| 166 | ST | *Leptodontidium orchidicola* strain UAMH56 | |  | 125 | | PP | *Ciboria asphodeli* strain F142282 | |  |
| 161 | SFY | *Capronia* sp. SL102031 | |  | 124 | | ST | *Plectania zugazae* MA Fungi 53068 | |  |
| 160 | ST | *Mortierella antarctica* strain MKOTU52 | |  | 120 | | ST | *Hygrocybe conica* PBM 918 isolate AFTOL-I | |  |
| 155 | SBR | *Ceratobasidium* sp. 3 RJ-2015 isolate 346 | |  | 118 | | EM | *Amphinema* sp. 554 VM-2015 | |  |
| 154 | PP | *Phoma* sp. MJW-2010e isolate VT 3-2-1-7r | |  | 115 | | ST | *Umbelopsis vinacea* strain Um 053 | |  |
| 149 | EM | *Hebeloma monticola* voucher UPS:SM6133 | |  | 113 | | noID | OTU_534 | |  |
| 148 | EM | *Helvella crispa* isolate KK2.2 | |  | 113 | | ST | *Sordaria tomento-alba* strain CBS 260.78 | |  |
| 145 | AP | *Hirsutella thompsonii* voucher ARSEF 2800 | |  | 108 | | noID | OTU_572 | |  |
| 143 | ST | *Myrothecium roridum* strain IFB-E091 | |  | 106 | | ST | *Mortierella alpina* strain CBS 696.70 | |  |
| 138 | ST | *Phialea strobilina* strain CBS 643.85 | |  | 104 | | L | *Placynthiella icmalea* | |  |
| 127 | UK | *Paraphoma fimeti* isolate S7 | |  | 103 | | EM | *Piloderma* sp. 18 RT-2012 isolate FFP431 | |  |
| 118 | noID | OTU_501 | |  | 101 | | ST | *Leptodontidium* sp. Sib5-8-1 | |  |
| 115 | AP | *Pochonia* sp. LC-2016 | |  | 100 | | ST | *Trechispora* sp. LISU178537 | |  |
| 113 | ST | *Dactylella oviparasitica* isolate 50 | |  | 98 | | ER | *Meliniomyces bicolor* isolate OTU_1 | |  |
| 112 | noID | OTU_338 | |  | 88 | | PP | *Devriesia shelburniensis* strain CBS 1158 | |  |
| 111 | noID | *Praetumpfia obducens* strain C56 | |  | 88 | | ST | *Geastrum fuscogleba* strain NY Trappe 950 ^‡^ | |  |
| 111 | ST | *Pseudodictyosporium wauense* NBRC 30078 | |  | 87 | | ST | *Fimetariella rabenhorstii* isolate 247J14 | |  |
| 108 | noID | OTU_483 | |  | 81 | | ST | *Coniochaeta* sp. XS62m1 | |  |
| 108 | noID | OTU_571 | |  | 77 | | PP | *Colpoma* sp. PDD 91607 | |  |
| 106 | MP | *Hypomyces completus* strain KSH411 | |  | 75 | | SFY | *Cladophialophora* sp. Leo15 | |  |
| 96 | noID | OTU_607 | |  | 73 | | EM | *Tomentella* sp. GO-2008-18 | |  |
| 95 | noID | OTU_326 | |  | 72 | | noID | OTU_704 | |  |
| 95 | noID | OTU_505 | |  | 72 | | PP | *Fusarium* sp. MX271 | |  |
| 94 | PP | *Ascochyta medicaginicola* var. *medicagini* | |  | 67 | | ST | *Clitopilopsis hirneola* strain CBS 126.46 | |  |
| 92 | noID | OTU_538 | |  | 62 | | EM | *Tricholoma arvernense* | |  |
| 92 | noID | OTU_616 | |  |  | |  |  | |  |
| 92 | noID | OTU_618 | |  |  | |  |  | |  |
| 91 | PP | *Spizellomyces plurigibbosus* voucher DAOM | |  |  | |  |  | |  |
| 87 | ST | *Trichoderma atroviride* strain RW4A2P | |  |  | |  |  | |  |
| 85 | noID | OTU_581 | |  |  | |  |  | |  |
| 84 | ST | *Cercophora coprophila* strain IFO 32091 1 | |  |  | |  |  | |  |
| 83 | ST | *Penicillium tularense* strain AS3.14006 1 | |  |  | |  |  | |  |
| 83 | PY | *Candida* sp. NCAIM Y.01956 | |  |  | |  |  | |  |
| 82 | AP | *Lecanicillium fusisporum* | |  |  | |  |  | |  |
| 82 | ST | *Mycena albidoaquosa* voucher 960 | |  |  | |  |  | |  |
| 82 | SFY | *Exophiala* sp. CPC 12173 | |  |  | |  |  | |  |
| 79 | noID | OTU_668 | |  |  | |  |  | |  |
| 79 | ST | *Paecilomyces marquandii* | |  |  | |  |  | |  |
| 77 | ST | *Polydesmia pruinosa* voucher TNS-F12764 | |  |  | |  |  | |  |
| 76 | SY | *Leucosporidium scottii* culture-collection | |  |  | |  |  | |  |
| 75 | PP | *Ilyonectria robusta* isolate EFA 184 | |  |  | |  |  | |  |
| 75 | ST | *Mycena rebaudengoi* voucher 861 | |  |  | |  |  | |  |
| 73 | PP | *Fusicladium cordae* CCF 3843 | |  |  | |  |  | |  |
| 73 | PP | *Venturia tremulae* var. *tremulae* strain C | |  |  | |  |  | |  |
| 73 | ST | *Articulospora proliferata* strain CCM F-1 | |  |  | |  |  | |  |
| 72 | EM | *Hebeloma dunense* voucher IK-H0307 | |  |  | |  |  | |  |
| 72 | ST | *Chloridium* sp. TMS-2011 voucher MSbale50 | |  |  | |  |  | |  |
| 71 | ST | *Myrothecium masonii* strain ATCC 24426.2 | |  |  | |  |  | |  |
| 70 | noID | OTU_669 | |  |  | |  |  | |  |
| 69 | PP | *Roseodiscus subcarneus* isolate D. Haelew | |  |  | |  |  | |  |
| 68 | ST | *Schizothecium carpinicola* CBS 228.87 | |  |  | |  |  | |  |
| 67 | EM | *Hebeloma subconcolor* voucher ZT:ZT3375 | |  |  | |  |  | |  |
| 67 | ST | *Myrothecium roridum* strain CGMCC 3.3682 | |  |  | |  |  | |  |
| 65 | noID | OTU_735 | |  |  | |  |  | |  |
| 65 | EM | *Wilcoxina rehmii* | |  |  | |  |  | |  |
| 65 | ST | *Myrothecium roridum* strain IFB-E008 | |  |  | |  |  | |  |
| 62 | L | *Phylliscum demangeonii* UPS Wedin 7241 | |  |  | |  |  | |  |
| 62 | MP | *Hypocrea* sp. KBS0814F | |  |  | |  |  | |  |
| 61 | ST | *Rhodotorula* sp. 10.10.L31 | |  |  | |  |  | |  |

* Data from coarse soil fractions were analyzed in constrained ordination models conditioned by respective significant confounding factors. Taxa are ordered by total read number across all sam-ples. Only taxa each representing more than 0.01% of total reads are listed. † UK, unknown func-tion; EM, ectomycorrhizal; AM, arbuscular mycorrhizal; PP, plant pathogen; ST, saprotroph; SY, saprotroph yeast; SFY; saprotroph facultative yeast; SBR, saprotroph brown rot; L, lichenized; SWR, saprotroph white rot; AP, animal parasite; ER; ericoid; MP, mycoparasite; EP, endophyte; SP, saprotroph pathogen; noID, fungal taxon not identified. ‡ Taxa not significantly associated with area in models not conditioned by confounding factors.

**Table S10:** Fungal sporocarp abundance for individual taxa and total species richness and abundance from the 2014 sporocarp surveys in jack pine transects, at Gateway Hill (M) and Fort Chipewyan (TR1) sites. Also shown across all transects, is whether taxa from the sporocarp survey matched taxa detected in soil DNA (Y = exact match, N = no exact match) using 454 pyrosequencing or Illumina sequencing. The number of taxa detected using soil DNA that match each sporocarp taxon to the genus level is shown.

|  |  | **Transects surveyed** | | | | | | |  | **Pyrosequencing** | |  | **Illumina sequencing** | |
| --- | --- | --- | --- | --- | --- | --- | --- | --- | --- | --- | --- | --- | --- | --- |
| **Trophic function**^†^ | **Fungal taxon** | **M2** | **M7** | **TR1P** | **TR1B2** | **Both M** | **Both TR1** | **ALL four** |  | **Exact match?** | **Number of genus-level matches** |  | **Exact match?** | **Number of genus-level matches** |
| ST | *Ascomycte* | 0 | 0 | 8 | 0 | 0 | 8 | 8 |  | N | 0 |  | N | 0 |
| ST | *Auriscalpium* cf. *vulgare* | 0 | 2 | 0 | 0 | 2 | 0 | 2 |  | N | 0 |  | N | 0 |
| ST | *Clitocybe* cf. *anisata* | 0 | 3 | 0 | 0 | 3 | 0 | 3 |  | N | 4 |  | N | 3 |
| ST | *Clitocybe* cf. *phyllophila* | 0 | 110 | 0 | 0 | 110 | 0 | 110 |  | Y | 4 |  | Y | 3 |
| ST | *Clitopilopsis* cf. *hirneola* | 0 | 0 | 63 | 7 | 0 | 70 | 70 |  | N | 0 |  | Y | 1 |
| ST | *Gymnopus* cf. *barbipes* | 4 | 6 | 0 | 0 | 10 | 0 | 10 |  | N | 2 |  | N | 1 |
| ST | *Gymnopus* cf. *dryophilus* | 1 | 1 | 0 | 0 | 2 | 0 | 2 |  | Y | 2 |  | Y | 1 |
| ST | *Hygrophorus* cf. *subalpinus* | 0 | 0 | 1 | 0 | 0 | 1 | 1 |  | N | 1 |  | Y | 1 |
| ST | *Lycoperdon* cf. *perlatum* | 1 | 0 | 0 | 0 | 1 | 0 | 1 |  | N | 2 |  | N | 1 |
| ST | *Mycena* cf. *pura* | 10 | 0 | 0 | 0 | 10 | 0 | 10 |  | Y | 5 |  | N | 3 |
| ST | *Mycena* sp. | 37 | 26 | 0 | 0 | 63 | 0 | 63 |  | Y | 5 |  | Y | 3 |
| EM | *Hebeloma* sp. | 3 | 0 | 0 | 0 | 3 | 0 | 3 |  | Y | 6 |  | Y | 1 |
| EM | *Hydnum* cf. *repandum* | 0 | 0 | 1 | 0 | 0 | 1 | 1 |  | N | 0 |  | N | 0 |
| EM | *Hygrophorus* cf. *agathosmus* | 0 | 1 | 0 | 0 | 1 | 0 | 1 |  | N | 1 |  | N | 1 |
| EM | *Inocybe* cf. *jacobi* | 0 | 0 | 8 | 1 | 0 | 9 | 9 |  | N | 12 |  | N | 10 |
| EM | *Inocybe* cf. *praetervisa* | 5 | 17 | 0 | 0 | 22 | 0 | 22 |  | N | 12 |  | N | 10 |
| EM | *Russula* cf. *renidens* | 0 | 0 | 10 | 0 | 0 | 10 | 10 |  | N | 5 |  | N | 1 |
| EM | *Thelephora* cf. *anthocephala* | 0 | 0 | 10 | 0 | 0 | 10 | 10 |  | N | 4 |  | N | 5 |
| EM | *Tricholoma* cf. *robustum* | 0 | 0 | 1 | 0 | 0 | 1 | 1 |  | N | 4 |  | N | 3 |
| ES | *Lyophyllum* cf. *decastes* | 0 | 1 | 0 | 0 | 1 | 0 | 1 |  | Y | 2 |  | Y | 4 |
| ES | *Ramaria* cf. *abietina* | 3 | 48 | 0 | 0 | 51 | 0 | 51 |  | Y | 1 |  | Y | 1 |
| ST | species richness | 5 | 6 | 3 | 1 | 8 | 3 | 11 |  | 4 | 8 |  | 5 | 9 |
| EM | species richness | 2 | 2 | 5 | 1 | 3 | 5 | 8 |  | 1 | 7 |  | 1 | 7 |
| ES | species richness | 1 | 2 | 0 | 0 | 2 | 0 | 2 |  | 2 | 2 |  | 2 | 2 |
| All | species richness | 8 | 10 | 8 | 2 | 13 | 8 | 21 |  | 7 | 17 |  | 8 | 18 |
| ST | sporocarp abundance | 53 | 148 | 72 | 7 | 201 | 79 | 280 |  |  |  |  |  |  |
| EM | sporocarp abundance | 8 | 18 | 30 | 1 | 26 | 31 | 57 |  |  |  |  |  |  |
| ES | sporocarp abundance | 3 | 49 | 0 | 0 | 52 | 0 | 52 |  |  |  |  |  |  |
| All | sporocarp abundance | 64 | 215 | 102 | 8 | 279 | 110 | 389 |  |  |  |  |  |  |

For mean richness and abundance per 100 m^2^ at each site, divide by 2; at all sites, divide by 4.

^†^ ST, saprotroph; EM, ectomycorrhizal; ES, ectomycorrhizal with saprotrophic capabilities.

**Figure S1:** Boxplots of pyrosequenced fungal richness (Observed) and α-diversity (Chao1, abundance-based coverage estimator [ACE], Shannon, Inverse Simpson [1/*D*], and Fisher indexes) in soil fractions from preliminary assessment of Fort Chipewyan road (forestry) and Gateway Hill (reclamation) sites. Each point represents one sample. For individual samples the Chao1 and ACE estimates also depict standard errors as defined by Chiu et al. ^a^ and O’Hara ^b^, respectively. The Shannon index was defined as *H =* -Σ *p_i_* ln(*p_i_*), where *p_i_* is the proportional abundance of species *i*. The inverse Simpson index was based on *D =* Σ *p_i_*^2^.

^a^ Chiu, C.H., Wang, Y.T., Walther, B.A., Chao, A.N., 2014. An improved nonparametric lower bound of species richness via a modified Good-Turing frequency formula. Biometrics 70, 671-682.

^b^ O’Hara, R.B., 2005. Species richness estimators: how many species can dance on the head of a pin? J. Anim. Ecol. 74, 375-386.

**Figure S2:** Principal coordinates of neighbour matrices (PCNM) representing spatial coordinates of stands sampled in the preliminary assessment. Depicted components were selected in models describing fungal communities in forest floor or mineral soil layers (bottom panels) and both soil layers jointly (top panels). Point size in plots is proportional to PCNM values.

**Figure S3:** Boxplots of pyrosequenced fungal community richness (Observed) and α-diversity (Chao1, abundance-based coverage estimator [ACE], Shannon, Inverse Simpson [1/*D*], and Fisher indexes) in soil fractions of jack pine transects at Fort Chipewyan road (forestry) and Gateway Hill (reclamation). Each point represents one sample. For individual samples the Chao1 and ACE estimates also depict standard errors as defined by Chiu et al. and O’Hara, respectively. The Shannon index was defined as *H =* -Σ *p_i_* ln(*p_i_*), where *p_i_* is the proportional abundance of species *i*. The inverse Simpson index was based on *D =* Σ *p_i_*^2^.

**Figure S4:** The effects of reclamation status, as depicted by partially constrained ordinations of the relative abundances and presence/absence of pyrosequenced fungal taxa (A and B, respectively), the relative abundance of fungal trophic functions (C), and the number of fungal taxa belonging to fungal trophic functions (D) in roots and coarse soils in jack pine transects at Gateway Hill (reclamation) and Fort Chipewyan road (forestry), while removing effects of soil fraction. Both species and sites scores represent unscaled raw eigenvalues. Polygons represent k-means clustering based on constrained axis.

**Figure S5:** The effects of reclamation status, as depicted by constrained ordinations of the relative abundances and presence/absence of Illumina-sequenced fungal taxa (A and B, respectively), the relative abundance of fungal trophic functions (C), and the number of fungal taxa belonging to fungal trophic functions (D) in coarse soils in jack pine transects at Gateway Hill (reclamation) and Fort Chipewyan road (forestry). Both species and sites scores represent unscaled raw eigenvalues. Polygons represent k-means clustering based on axes 1 and 2.
